# The self-selected intensity of physical activity during real-life e-bike commuting

**DOI:** 10.3389/fspor.2025.1653833

**Published:** 2026-01-13

**Authors:** Amund Riiser, Eivind Aadland, Solveig Nordengen

**Affiliations:** Department of Sport, Food and Natural Sciences, Faculty of Education, Arts and Sports, Western Norway University of Applied Sciences, Sogndal, Norway

**Keywords:** electric bikes, pedelec, active travel, health, physical activity

## Abstract

**Background:**

Decreasing physical activity levels present a major public health challenge. The use of e-bikes has risen substantially over the past decade, presenting a potential solution to common barriers associated with physical activity and conventional cycling. However, the intensity of e-bike commuting in real-life settings remains unknown. This study aimed to investigate the self-selected intensity and the impact of topography on intensity during regular e-bike commuting.

**Methods:**

In this cross-sectional study, oxygen consumption, power output, heart rate, perceived exertion, and positional data were recorded from 19 commuters [mean (standard deviation) age 41 (8) years] during their regular commutes on their own e-bikes. Data were summarized and analyzed in 10, 30, and 60 s epochs, and adjusted for the duration of the commutes. Intensity of the commutes was compared between downhill, flat, or uphill segments using linear mixed models. The intensity of physical activity was defined as light <3 metabolic equivalents (METs), moderate 3–5.9 METs, or vigorous ≥6 METs.

**Results:**

Oxygen consumption during e-bike commuting was mean (standard deviation) 20.8 (5.0) mL/kg/min [5.9 (1.4) METs]. Depending on epoch lengths, 44%–48% of the commutes were classified as vigorous physical activity. Across epoch lengths, the mean intensity of the commutes was classified as moderate (4.6–4.8 METs) during downhill, moderate (5.5–5.9 METs) during flat, and vigorous (7.0–7.5 METs) during uphill riding (*p* < 0.001).

**Conclusions:**

Our findings suggest that e-bike commuters self-selected moderate to vigorous intensities during real-life commutes, aligning with international physical activity guidelines for improving public health. Therefore, policies promoting a shift from car use to e-biking could have significant public health benefits.

## 1 Introduction

There is a dose-response relationship between the intensity, duration, and frequency of physical activity and physical and mental health (1). However, 31% of adults globally do not meet the WHO physical activity recommendations, and despite goals to reduce global physical inactivity levels by 15% from 2010 to 2030 (2), inactivity levels continue to rise (3). A common barrier to regular physical activity is a lack of time (4); thus, integrating physical activity into daily routines through active transportation, such as cycling, presents a practical solution. Cycling for transport is generally associated with moderate-to-vigorous intensity and is well-documented to reduce all-cause mortality (5), cardiovascular disease (6), diabetes (7), and other health issues (8). However, barriers such as long distances and hilly terrain limit its adoption (9).

Pedal-assisted bicycles (e-bikes), which integrate an electric motor and battery to support pedalling, have become very popular in the USA and Europe over the past decade (10). E-bike users tend to commute longer distances and more frequently than traditional bicycle users (11), highlighting e-bikes' potential to increase daily physical activity levels if commuters switch from car or public transportation to e-bike commuting. The use of e-bikes is often motivated by overcoming common barriers to conventional commuter cycling, including hilly terrain (12). However, the effect of topography on exercise intensity while commuting with e-bike has not been investigated.

Several studies have explored the exercise intensity of e-biking. However, these studies often lack measures of power output and oxygen consumption (VO_2_) (13, 14) and use experimental designs with unfamiliar bikes and fixed assistance levels on predefined routes (15–23). This raises questions about the generalizability of these findings to real-life commuting, where riders use familiar bikes on regular routes and perform tasks such as braking, shifting gears, and adjusting assistance levels with varying topography (24–26). Familiarity with both the bike and the route likely influences the riding behaviour and the resulting physical activity intensity (26).

The intensity of physical activity can be expressed as absolute or relative. Absolute intensity is often quantified using the metabolic equivalent (MET), which measures energy expenditure relative to VO_2_ at rest (3.5 mL/kg/min) (27).

Global physical activity guidelines categorize physical activity into light physical activity (LPA, 1.5–2.9 METs), moderate physical activity (MPA, 3.0–5.96 METs), or vigorous physical activity (VPA, ≥6.0 METs), and recommend that adults engage in at least 150–300 min of moderate-to-vigorous physical activity (MVPA) or 75–150 min of VPA weekly for substantial health benefits (1). Alternatively, relative intensity refers to the workload measured as a proportion of an individual's maximal aerobic capacity (28), which is often used in exercise studies. While absolute intensity is independent of maximal aerobic capacity, relative intensity will vary largely depending on the aerobic capacity of an individual. Thus, these definitions might classify intensity of physical activity differently.

In a recent review, Jenkins and colleagues (29) emphasized the need for research on self-selected intensity during real-life e-bike commuting. This knowledge is critical to estimate potential public health effects and guide recommendations on e-bike use. Therefore, the present study aimed to investigate the self-selected intensity and the effect of topography on intensity levels among regular e-bike commuters during their daily commutes.

## 2 Methods

This cross-sectional study was conducted in Norway during May and June 2024. We recruited a convenience sample of 19 e-bike commuters (9 males, 10 females) with a mean (standard deviation, SD) age of 41 (8) years. Participants were recruited through personal networks, flyers distributed at bicycle parking areas, and by directly approaching e-bike commuters. Inclusion criteria required participants to regularly commute by e-bike to a university campus area hosting schools, business companies and a university, and to use the e-bike they normally use for commuting. Eligible e-bikes complied with Norwegian technical specifications: 1) The motor assists only when pedalling at speeds <25 km/h, 2) The motor can assist without pedalling up to 6 km/h, and 3) The motor's nominal power does not exceed 0.25 kW.

Participants completed an online survey (SurveyXact by Ramboll). The survey (Supplementary File) was developed for the present study and provided details about their age, gender, height, weight, year of e-bike acquisition, rating of perceived exertion (RPE) during commuting [CR-10 scale with verbal anchors (30)] in addition to perceived advantages of e-bike commuting, commuting habits, and commuting habits before starting e-bike commuting (in preselected statements).

The commute was conducted on separate days for each participant and in dry conditions with temperatures above 15 °C due to specifications of the VO_2_ analyser. Participants were instructed to follow their usual route home at their typical commuting intensity. They were instructed to avoid stopping for grocery shopping, picking up children at the kindergarten etc. during the commute. Intensity of physical activity was assessed with different measures during one commute from the campus to the participants' residence. These measures were fitted to person/bike by the study staff, except the HR strap that was fitted by the participant under instruction from the study staff.

Oxygen consumption (VO_2_) (mL/kg/min) was measured using a portable oxygen analyser (VO_2_ Master, Health Sensors Inc., Vernon, British Columbia, Canada), calibrated before each commute with a 3-liter syringe according to the manufacturer guidelines. Participants wore an over-nose mask (Hans Rudolph, Inc., Shawnee, KS, USA) fitted with a “user piece” designed for ventilation ranging from 30 to 150 L/min. Heart rate (beats per minute), distance (meters), speed (km per hour), duration (seconds), and elevation (meters above sea level) was recorded using a Garmin Edge 1030 Plus GPS device (Garmin, Olathe, KS, USA), connected to a chest strap (Garmin, Olathe, KS, USA) for heart rate monitoring. Maximal heart rate (HR_max_) was predicted by the equation 211 − 0.64·age (31). Change in elevation was computed from the sampled data file as the difference in elevation between each measurement and the previous measurement. The topography was classified as downhill, flat or uphill defined as the first, second, and third tertile of change in elevation.

Pedal power was measured using dual-sided power pedals (Garmin Rally XC200, Olathe, KS). As all participants normally used platform pedals, we used commercially available plastic platform pedal adapters fitted in the slots for cleats (normally attached to cycling shoes). The pedals were manually calibrated according to the manufacturer's instructions each time they were fitted to an e-bike.

Data from the oxygen analyser, chest strap and power pedals were synchronized with the GPS device. In addition to measured VO_2,_ we predicted VO_2_ using the American College of Sports Medicine formula for cycling power conversion: “VO_2_ = [1.8 (kilogram-force meter/minute) ÷ body mass in kg] + 7” (32). Kilogram-force meter/minute was converted from watts by multiplying by 6.

In primary analyses, we expressed physical activity intensity as multiples of resting energy expenditure (LPA: 1.5–2.9, MPA: 3.0–5.9 METs and, and VPA: ≥6.0 METs) based on commonly applied definitions (28, 33). In secondary analyses, we assessed the intensity of physical activity relative to each individual's estimated HR_max_ (LPA: 40 < 55% of HR_max_, MPA: 55 < 70% of HR_max_ and VPA: 70 < 90% of HR_max_ (28).

### 2.1 Statistical analysis

Data from the commute was averaged into epochs of 10, 30, or 60 s or the whole commute to investigate whether data resolution influenced the results.

Data are presented as means (SD) unless stated otherwise.

To adjust for differences in numbers of epochs between participants due to different durations of the commutes, the proportion of LPA, MPA, and VPA was calculated for each participant and presented as mean values for the total sample. The difference in intensity of commuting between types of topography was assessed with a linear mixed model including random intercepts for participants given inclusion of multiple observations for each participant. Topography was included as the independent variable, whereas measures of intensity (VO_2_, power and heart rate) were included as dependent variables in separate models. Linear mixed models with random intercepts for participants were also used to assess the association between VO_2_ (dependent variable) and change in elevation (independent variable). All associations were reported as standardized coefficients (*β*) from these models. Pairwise comparisons between downhill, flat and uphill segments were performed using Bonferroni correction for multiple testing.

A *p*-value < 0.05 was considered statistically significant. All analyses were performed using IBM SPSS v. 29.0.2.0 (IBM SPSS Statistics for Windows, Armonk, NY; IBM Corp., USA).

## 3 Results

### 3.1 Descriptive data of commuters and commutes

We recruited 19 participants (10 women and 9 men) aged 32–62 years. Characteristics of the participants and their commutes are provided in Table 1.

**Table 1:** Descriptive data from 19 regular e-bike commuters performing one commute from work to residence.

Descriptive variable	All (*n* = 19)	Women (*n* = 10)	Men (*n* = 9)
Age (years)	41 (8)	39 (4)	43 (10)
Height (cm)	173 (6)	169 (3)	178 (5)^b^
Weight (kg)	71 (11)	64 (9)	79 (9)^b^
Body mass index (kg/m^2^)	23.7 (2.7)	22.6 (2.7)	24.9 (2.3)
E-bike ownership (years)	4.6 (2.0)	5.1 (1.9)	4.0 (2.1)
VO_2_ (mL/kg/min)	20.8 (5.0)	20.3 (5.9)	21.4 (3.9)
Power (watt/kg)	1.36 (0.34)	1.36 (0.37)	1.35 (0.33)
Heart Rate (%max)	60 (7)	62 (6)	58 (9)
Rating of perceived exhaustion (1–10)	3.4 (1.2)	3.10 (1.1)	3.8 (1.3)
Distance (m)^a^	3,989 (1,712)	4,278 (1,500)	3,627 (1,988)
Speed (km/h)	21.5 (3.1)	22.5 (2.9)	20.4 (3.3)
Duration (min.sec)^a^	10.42 (4.03)	11.09 (0.29)	10.09 (4.51)
Total ascent (m)^a^	91.3 (31.8)	84.6 (17.9)	99.6 (43.5)
Total descent (m^a^)	49.5 (25.0)	55.5 (26.8)	42.0 (21.8)

All values are means (standard deviations).

a One participant rode 17,180 m and was omitted from the analyses of distance, duration, assent and decent to avoid a skewed sample.

b Statistically significantly different from women. Maximal oxygen consumption; VO_2._.

### 3.2 The intensity of physical activity during commuting

The mean (SD) VO_2_ of commuting was 20.8 (5.0) mL/kg/min, corresponding to 5.9 (1.4) METs. Based on the absolute definition of intensity (i.e., METs above resting values), irrespective of epoch length, <10% of the commute was classified as LPA, >46% of the commute as MPA and >44% of the commute as VPA (Table 2). In our secondary analysis using a relative intensity definition (i.e., % of HR_max_), 31/30/29% of the commute was classified as LPA, 51/50/51% as MPA, and 15/16/16% as VPA for epoch lengths of 10/30/60 s, respectively. The remaining 3/4/4% of the commute was performed with a heart rate <40% HR_max_.

**Table 2:** The proportion of time spent in different categories of absolute intensity of physical activity (PA) during commuting with e-bike.

Epoch	Light PA (1.5–2.9 METs)	Moderate PA (3.0–5.9 METs)	Vigorous PA (≥ 6.0 METs)
10 s	9.4%	46.4%	44.8%
30 s	5.9%	45.9%	48.2%
60 s	5.7%	48.3%	47.3%

The data is based on measured oxygen consumption and converted to metabolic equivalents (METs).

The mean absolute exercise intensity of riding downhill and flat corresponded to MPA (3.0–5.9 METs) and uphill riding corresponded to VPA (≥6.0 METs). The absolute and relative intensity of physical activity differed across the topography types, being higher (*p* < 0.001) for uphill than for flat topography and higher (*p* < 0.001) for flat than for downhill topography. Different epoch lengths did not affect the assessment of intensity (Table 3). Adjusted for multiple observations from the participants, VO_2_ was associated with change in elevation assessed with 60 (standardized *β* = 0.529) 30 (standardized *β* = 0.507) and 10 (standardized *β* = 0.402) second epochs (*p* < 0.001) (Supplementary Figure S1).

**Table 3:** Oxygen consumption (VO_2_), power output and heart rate (HR) during e-bike commuting downhill, flat and uphill.

Epoch	VO_2_, downhill	VO_2_, flat	VO_2_, uphill	Power, downhill	Power, flat	Power, uphill	HR, downhill	HR, flat	HR, uphill
10 s	16.67 (6.03)	^a^19.87 (6.87)	24.56 (7.38)	0.86 (0.87)	^a^1.34 (0.83)	1.92 (0.76)	53 (8)	^a^60 (10)	67 (10)
30 s	16.17 (5.47)	^a^20.57 (6.28)	25.01 (6.96)	0.74 (0.65)	^a^1.35 (0.71)	1.89 (0.68)	53 (7)	^a^60 (10)	67 (10)
60 s	16.66 (5.36)	^a^19.15 (5.58)	26.16 (6.24)	0.72 (0.6)	^a^1.34 (0.65)	1.85 (0.69)	54 (8)	^a^59 (9)	67 (10)

a = statistically significantly different from uphill and downhill (*p* < 0.001). Units: VO_2_; mL/kg/min, Power output; watt/kg, HR; % of estimated maximal heart rate.

Based on mean data from the whole commute, we investigated the associations between the different measures of intensity of physical activity shown in Table 1. We found positive associations between VO_2_ and power (standardized *β* = 0.663, *p* = 0.002) and between RPE and HR (standardized *β* = 0.530, *p* = 0.024), whereas other associations were non-significant. There was no difference (*p* = 0.265) between measured VO_2_ [20.8 (5.0) mL/kg/min] and predicted VO_2_ [21.7 (3.7) mL/kg/min].

### 3.3 Commuting habits

In the online survey, 10, 16, and 74% of the participants reported that they commuted with e-bike <2 days, 2–4 days, and >4 days a week, respectively. Before the participants acquired an e-bike, the most common ways of commuting <2 days, 2–4 days, and >4 days a week were by conventional bike (11, 16, 32%, respectively) or by car (11, 32, 26%, respectively).

The perceived advantages of e-bike commuting “Cheaper than the previous means of transportation”, “Environmentally friendly” and “Faster than walking” were reported by 74% of the participants, while “Don't spend time looking for parking space”, “It's fun”, and “Good exercise” was reported by 68%, 47%, and 37% of the participants, respectively.

## 4 Discussion

The present study investigated the intensity of physical activity during real-life e-bike commuting. Our findings indicate that e-bike commuting was performed with a mean VO_2_ of 20.8 mL/kg/min, corresponding to 5.9 METs, which classifies as moderate to vigorous intensity (33). As the mean duration of the one-way commute was 10 min, 5 days of commuting by e-bike would contribute to 2/3 of the minimum 150 min of MVPA per week recommended by the WHO for this group. Additionally, when factoring in that 45% (or more) of the commute consisted of VPA, for which guidelines recommend a minimum of 75 min per week as an alternative to 150 min per week of MVPA, and 10% (or less) is LPA, a dedicated e-bike commuter in this sample could accumulate 90% of the minimum recommended physical activity while commuting.

In our study, e-bike commuting was performed at an average absolute intensity of 5.9 METs, categorized as MVPA. This intensity aligns with results presented in the systematic review by McVicar et al. (34), reporting self-selected intensities ranging from 5.1 METs to 8.5 METs on preselected routes with moderate electrical assistance. Compared to our study, higher intensities, approaching VPA, have been reported in different populations: Berntsen et al. (16) reported a median of 8.5 METs in active adults, Gojanovic et al. (17) reported a mean of 7.3 METs in sedentary adults, and La Salle et al. (21) reported a mean of 8.3 METs in young adults. However, lower values (5.1 METs) (35) and similar values (6.1 METs) (23), have also been reported. The mean power output recorded in our study was 1.36 W/kg (96 W), comparable to Simons et al.'s (23) report of 94 W in a sample of adults aged 53 years. This finding indicates reliable measurements as both power output and VO_2_ measurements are comparable in the two studies. However, direct comparisons to other studies are challenging due to variations in study designs. We evaluated real-life commutes, while others simulated commutes with fixed routes and supplied bikes. Thus, we argue that our study adds ecological validity to previous studies.

Our study further demonstrates that route topography significantly affected exercise intensity during commutes. Uphill segments were ridden with vigorous intensity compared to flat topography performed with moderate intensity. This is interesting since the motivation for using e-bikes often is the e-bikes' ability to overcome terrain barriers (12). Our finding is consistent with findings from simulated commutes on hilly routes (Langford et al. (35)), and indicates that the increased intensity when commuting in hilly topography may provide even more health benefits compared to commuting in flat topography.

The mean commuting distance in our study was 4 km (one way), aligning with the mean daily distances reported in the review by Bourne et al. (12) (3–12 km). In addition, we found that e-bike commuting mostly replaced traditional bike commuting and car commuting, also consistent with previous research (12). The similarities in commuting behaviour between our sample and other samples may suggest that our findings are representative of typical e-bike commutes and thus may generalize to a wider population.

Depending on epoch length, we found that 91%–96% of the commutes were classified as MVPA when using an absolute definition of intensity (i.e., METs), while 66%–67% of the commutes was classified as MVPA when using a relative definition of intensity (i.e., % HRmax). A systematic review of the agreement between absolute and relative intensity of physical activity during walking reported that there was agreement in 43% of all trials (categorized mean intensity) (36). However, the comparison between trials is challenging as multiple measures of relative intensity (as %HR_max_, %VO_2max_, %HR_reserve_ and %VO_2reserve_,) were used, in addition to application of different cut-off points for HR_max_ (28). The use of METs and heart rate to categorize physical activity intensity in epidemiological studies has both advantages and limitations. Whereas METs provide an objective measure of energy expenditure, % HR_max_ takes each individual's fitness level into account (36). We did not assess aerobic fitness level in the present study. However, our participants had normal bodyweight (BMI < 25) while the BMI in a representative sample of Norwegian population is >25 and categorized as overweight (31). Our participants also commuted to a university campus where most employees have higher education. Both factors indicate a higher fitness level in our sample than in the general population. As aerobic fitness is associated with heart rate (during rest and work) (37), we expect that the difference between the two classification methods may have been smaller in a less fit population.

As VO_2_ is a response to increased power output when cycling, the increase in VO_2_ will follow from the increase in power (38). However, while power output may change second-by-second, VO_2_ will lag and average over longer time periods. Thus, we investigated the proportion of time spent in different intensity categories during commuting and the association between topography and intensity of physical activity using epoch lengths of 10, 30, and 60 s. We found that the use of different epoch lengths provided no meaningful difference to our results. These findings suggest that future studies investigating the distribution of physical activity intensity during e-bike commuting may use epoch lengths up to one minute without losing valuable information, though this may depend on research questions and context.

The growing popularity of e-bikes over the past two decades (10) and the level of MVPA during e-bike commuting suggest that e-biking can be valuable for increasing physical activity levels and improving public health (1). While e-biking is performed at lower intensity than conventional bicycling (34), its potential benefits are only achieved when it replaces more passive forms of commuting such as car travel or public transportation. A scoping review reports that e-bike ownership can replace 20%–86% of car trips and 23%–72% of conventional bike trips (12). Even if e-biking can lead to significant health benefits, the increased popularity of e-biking has also led to an increase in e-bike related injuries (39), which should also be taken into account to better inform the public and policymakers about its potential pros and cons.

### 4.1 Strengths and limitations

The ecological validity of our study is a key strength, as it measured absolute physical activity intensity during actual e-bike commutes, rather than simulated conditions as previous studies have done. Our study is strengthened by measurement of VO_2_ and power. In contrast, most studies within the epidemiology of physical activity use algorithms and accelerometer data to assess the intensity of physical activity. Thus, the direct measurement of VO_2_ provided a reliable basis for calculating METs compared to most other studies in the field. Although our portable oxygen analyser has shown considerable day-to-day variation in VO_2_ measurements (40), it exhibited no difference between indoor and outdoor measurements (41). While our oxygen analyser has not been extensively validated, our measured VO_2_ values were not statistically different to the VO_2_ values predicted using the power values, increasing our confidence in the results.

Nonetheless, several limitations should be acknowledged. Although the ecological validity attained from real-life commuting and gold standard measurements are major strengths of the present study, ecological validity could be limited by several factors. Due to the operating specifications of the analyser, VO_2_ measurements could only be performed in dry conditions and in ambient temperatures above 15 ° Celsius. Thus, all measurements were performed in May and June during dry conditions, pleasant temperatures and daylight. Commuting during cold and wet weather, or limited daylight was not observed. Neither did we investigate the level of assistance for the motor, or physiological and motivational factors. To our knowledge, no studies have investigated the effect of these factors on commuting intensity, and it is not clear how they affect self-selected intensity. While the intensity may be reduced during less comfortable conditions, due to increased caution in dark or in slippery conditions or when wearing heavy clothing, these factors may as well increase intensity due to increased rolling resistance (in snow) and increased workload. Importantly, in contrast to recreational bicycling, the commuting distance, and thus the workload, will be rather constant. The frequency of commuting is reduced in less favourable weather conditions (12), which will lead to fewer minutes of physical activity. As discussed earlier, our sample may also be fitter than the general population, and if commutes are performed at the same relative intensity irrespective of fitness levels, the MET values may be overestimated in our sample compared to a broader population sample. Our small convenience sample may not be representative of the broader Norwegian e-bike commuting population, and most commutes were conducted in the same area. Our university campuses are located in urban areas, but with relatively little traffic compared to many larger cities. E-biking in a more traffic dense environment may lead to more stops at intersections and a lower speed, and thus a lower mean intensity. Norway, and particularly the areas where the study was performed, are characterised by hilly terrain. Our findings indicate that uphill e-biking is associated with VPA, whereas riding on flat terrain primarily corresponds to MPA. This suggests that in environments characterized by less elevation, e-biking may contribute more to MPA than VPA. Furthermore, measuring only one commute per participant may introduce a reactive effect where participants alter their behaviour due to being observed. We sought to limit change in behaviour by instructing participants to commute normally and leveraging the familiarity of their usual routes and bikes, as opposed to using predefined routes and unfamiliar bikes as in other studies. Our primary analysis was based on measurements of absolute intensity, METs. Still, measurement of HR_max_ and maximal VO_2_ would have been helpful in our secondarily analysis determining relative intensity of physical activity. We estimated HR_max_ using an equation based on a large sample of adults at approximately the same age and the same nationality. However, even if HR_max_ is linearly reduced with age, the prediction of HR_max_ has a standard error of 10.8 beats per minute making such estimations challenging (31). Still, such individual variations would minimally affect group level estimates and only our secondary findings used relative intensity.

Due to practical reasons related to the measurements, we instructed the participants to ride from work directly to their home without any stops for grocery shopping, picking up children, etc. Thus, we did not measure the intensity of physical activity during these behaviours. The ability to carry heavy loads is a commonly reported benefit of e-biking (12), indicating that our participants occasionally would ride with extra loads. However, several studies report no difference in intensity of physical activity when riding an e-bike with or without extra load (20, 42). Further research is needed to investigate the intensity of physical activity during use of e-bike for transport in other populations, in particular in youths, in populations with low physical fitness levels and in commuters in complex traffic environments.

## 5 Conclusion

Our findings suggest that e-bike commuting can be classified as moderate to vigorous physical activity, the intensity of physical activity recommended by the WHO to improve public health. Thus, regular e-bike commuting can comprise a large part of the recommended weekly physical activity. These results suggest that the recent trend of increased popularity of e-bikes for commuting likely lead to significant health benefits. Therefore, measures to facilitate a switch in transport mode from cars to e-bikes may improve public health as well as reduce greenhouse gas emission and traffic density.

## Data Availability

The raw data supporting the conclusions of this article will be made available by the authors, without undue reservation.
