# Supplementary material for: The self-selected intensity of physical activity during real-life e-bike commuting
Source: Front Sports Act Living. 2026 Jan 13;7:1653833. doi: 10.3389/fspor.2025.1653833 (PMC12834821; doi:10.3389/fspor.2025.1653833)
Supplement: Supplementary file 1 [file Supplementaryfile1.docx]

**Fill in the participant ID. You can find this in the email you received along with this questionnaire.
If you are unsure, contact Amund (phone 926 88 239).**_____

By participating and providing answers from you and other participants, we will gain increased knowledge about the self-selected intensity of e-biking. In addition, we will gain a better understanding of the health benefits of e-bikes for adults.
You are of course allowed to skip individual questions. However, it is important that you provide honest answers. The information in this questionnaire is treated confidentially and your name will not appear in data files or written material.
It takes about 5 minutes to complete the questionnaire. Please follow the instructions along the way.
Thank you in advance for your help.

**Gender**
(1) Female
(2) Male
(3) Other

**What year were you born?**
E.g., 1985.

**How tall are you (cm)?**

**How many kilos do you weigh?**

**What is the highest level of education you have completed?**
(1) Less than 7 years of primary school
(2) Primary school 7-10 years, continuation school or folk high school
(3) Secondary school, middle school, vocational school, 1-2 years of high school
(4) High school diploma, economic high school, general studies in high school
(5) College/university, less than 4 years
(6) College/university, 4 years or more

**What is your main activity?**
(1) Full-time employed
(2) Part-time employed
(3) Unemployed
(4) Homemaker
(5) Retired/disability
(6) Student

**Approximately what was the household's total gross income last year? Include all income from work, benefits, social assistance, etc.**
E.g., 850000

**What year did you buy an e-bike?**
E.g., 2020.

**What advantages do you see in cycling to work/studies with an e-bike?**
(1) It is cheaper than the previously used means of transport
(2) It is environmentally friendly
(3) It is good exercise
(4) It is fun
(5) It is faster than walking
(6) No need to spend time finding parking
(7) None
(8) Other: _____

**How often did you use the following means of transport to get to and from work/studies before you had access to the e-bike?**

|  | More than 4 times a week | 2-4 days a week | 1 days a week | 1-3 days a month | Less often | Never |
| --- | --- | --- | --- | --- | --- | --- |
| Regular bike | (1) 🔾 | (2) 🔾 | (3) 🔾 | (4) 🔾 | (5) 🔾 | (6) 🔾 |
| Car | (1) 🔾 | (2) 🔾 | (3) 🔾 | (4) 🔾 | (5) 🔾 | (6) 🔾 |
| Public transport | (1) 🔾 | (2) 🔾 | (3) 🔾 | (4) 🔾 | (5) 🔾 | (6) 🔾 |
| Walking all the way | (1) 🔾 | (2) 🔾 | (3) 🔾 | (4) 🔾 | (5) 🔾 | (6) 🔾 |
| Working/studing at home | (1) 🔾 | (2) 🔾 | (3) 🔾 | (4) 🔾 | (5) 🔾 | (6) 🔾 |

**How often do you use the following means of transport to get to and from work/studies after you had access to the e-bike?**

|  | More than 4 times a week | 2-4 days a week | 1 days a week | 1-3 days a month | Less often | Never |
| --- | --- | --- | --- | --- | --- | --- |
| E-bike | (1) 🔾 | (2) 🔾 | (3) 🔾 | (4) 🔾 | (5) 🔾 | (6) 🔾 |
| Regular bike | (1) 🔾 | (2) 🔾 | (3) 🔾 | (4) 🔾 | (5) 🔾 | (6) 🔾 |
| Car | (1) 🔾 | (2) 🔾 | (3) 🔾 | (4) 🔾 | (5) 🔾 | (6) 🔾 |
| Public transport | (1) 🔾 | (2) 🔾 | (3) 🔾 | (4) 🔾 | (5) 🔾 | (6) 🔾 |
| Walking all the way | (1) 🔾 | (2) 🔾 | (3) 🔾 | (4) 🔾 | (5) 🔾 | (6) 🔾 |
| Working/studing at home | (1) 🔾 | (2) 🔾 | (3) 🔾 | (4) 🔾 | (5) 🔾 | (6) 🔾 |

**When you cycle with an e-bike, how strenuous do you normally feel a trip home from work/studies is?**
(1) 0 No effort at all
(2) 1 Very easy. Like a healthy person taking a short walk at their own pace.
(3) 2
(4) 3 Moderate is somewhat, but not particularly strenuous. It feels okay and it's not a problem to continue.
(5) 4
(6) 5 The work is strenuous and tiring, but you don't have major difficulties continuing. The effort is about half of "maximum".
(7) 6
(8) 7 Very strenuous, a very strong strain. You can continue, but you have to push yourself hard and you are very tired.
(9) 8
(10) 9
(11) 10 An extremely high level. It is the hardest most people have ever experienced before
