# Supplementary material for: The self-selected intensity of physical activity during real-life e-bike commuting
Source: Front Sports Act Living. 2026 Jan 13;7:1653833. doi: 10.3389/fspor.2025.1653833 (PMC12834821; doi:10.3389/fspor.2025.1653833)
Supplement: Supplementary file 2 [file Image1.pdf]

Supplementary figure 1a

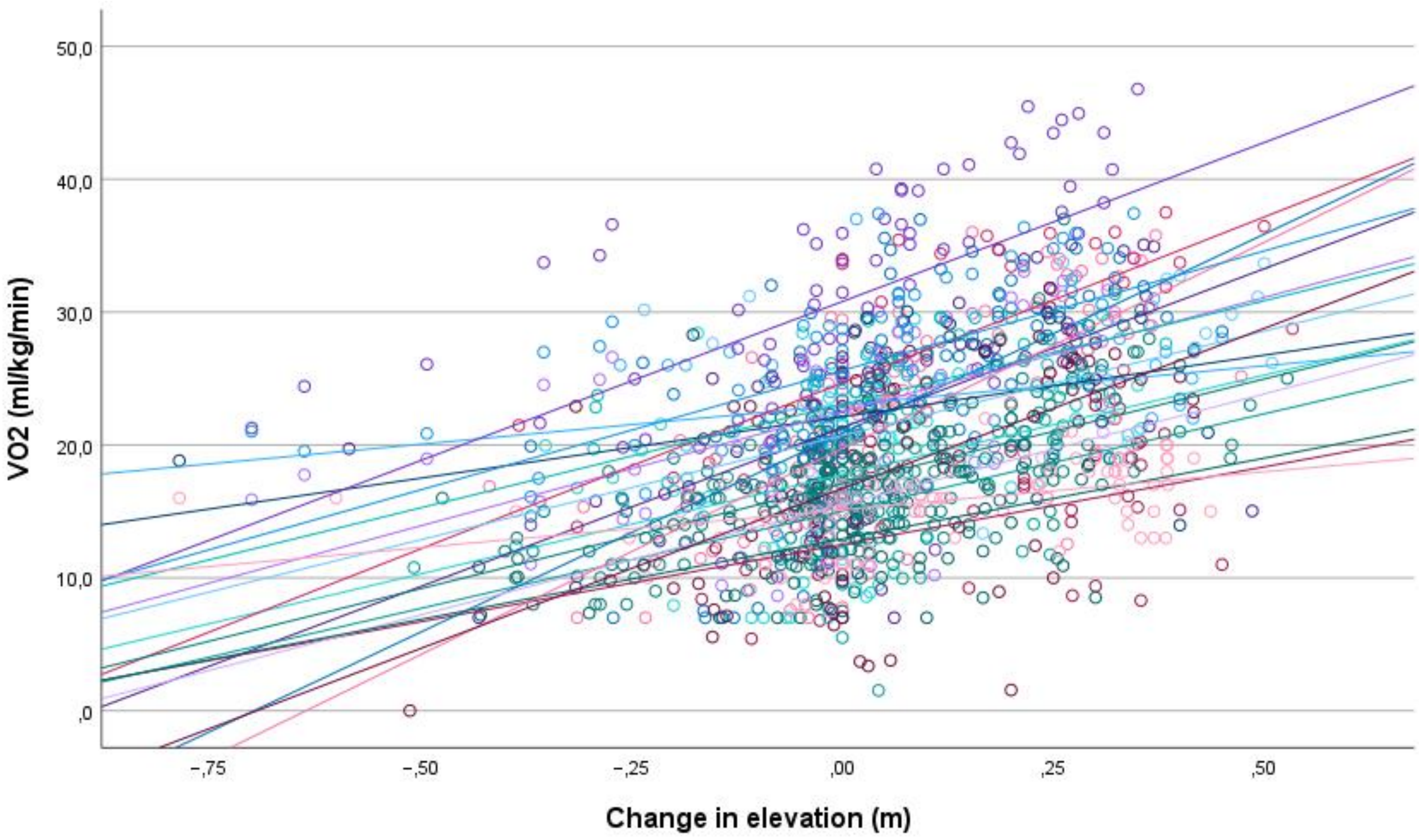

Supplementary figure 1b

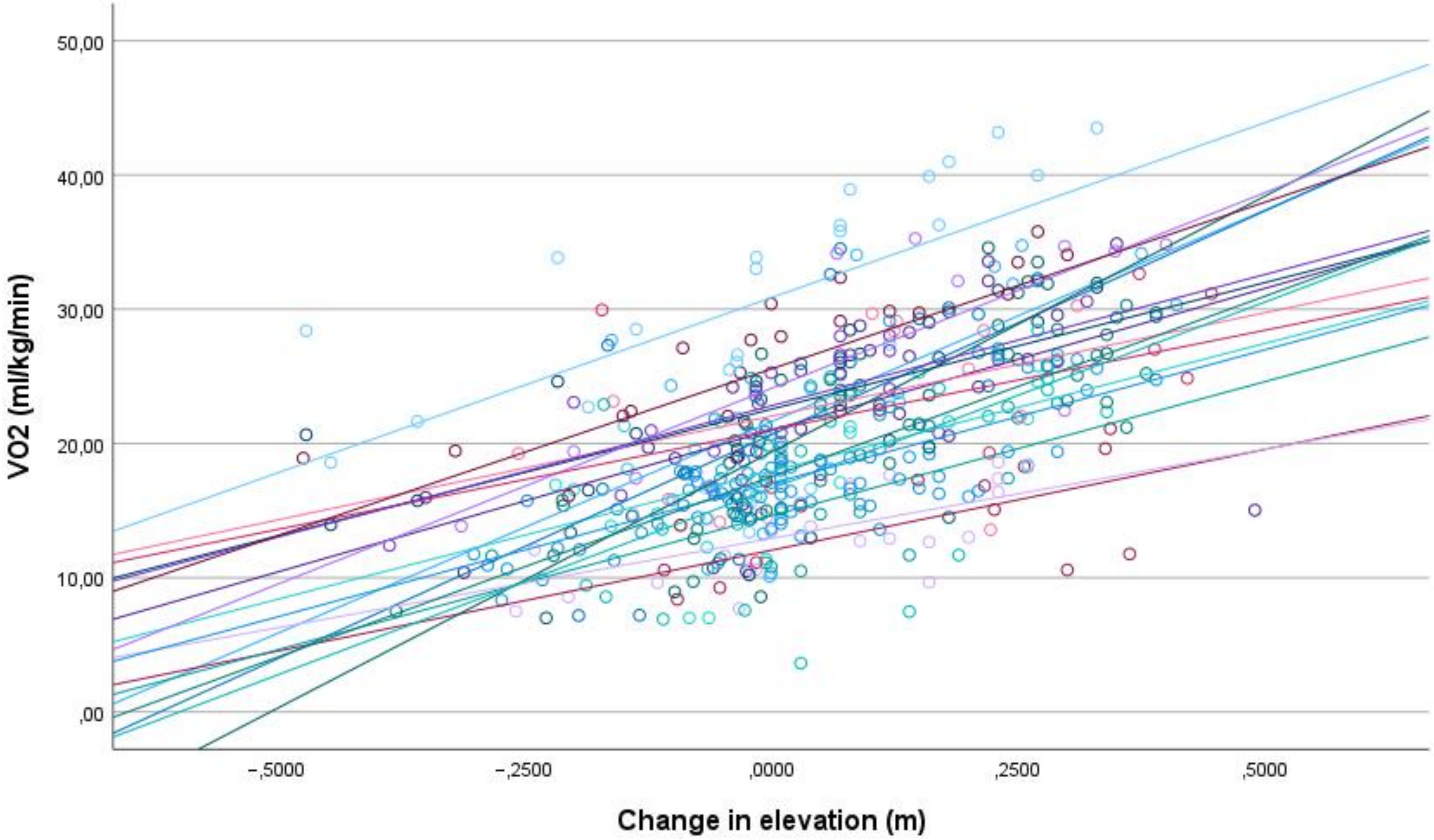

Supplementary figure 1c

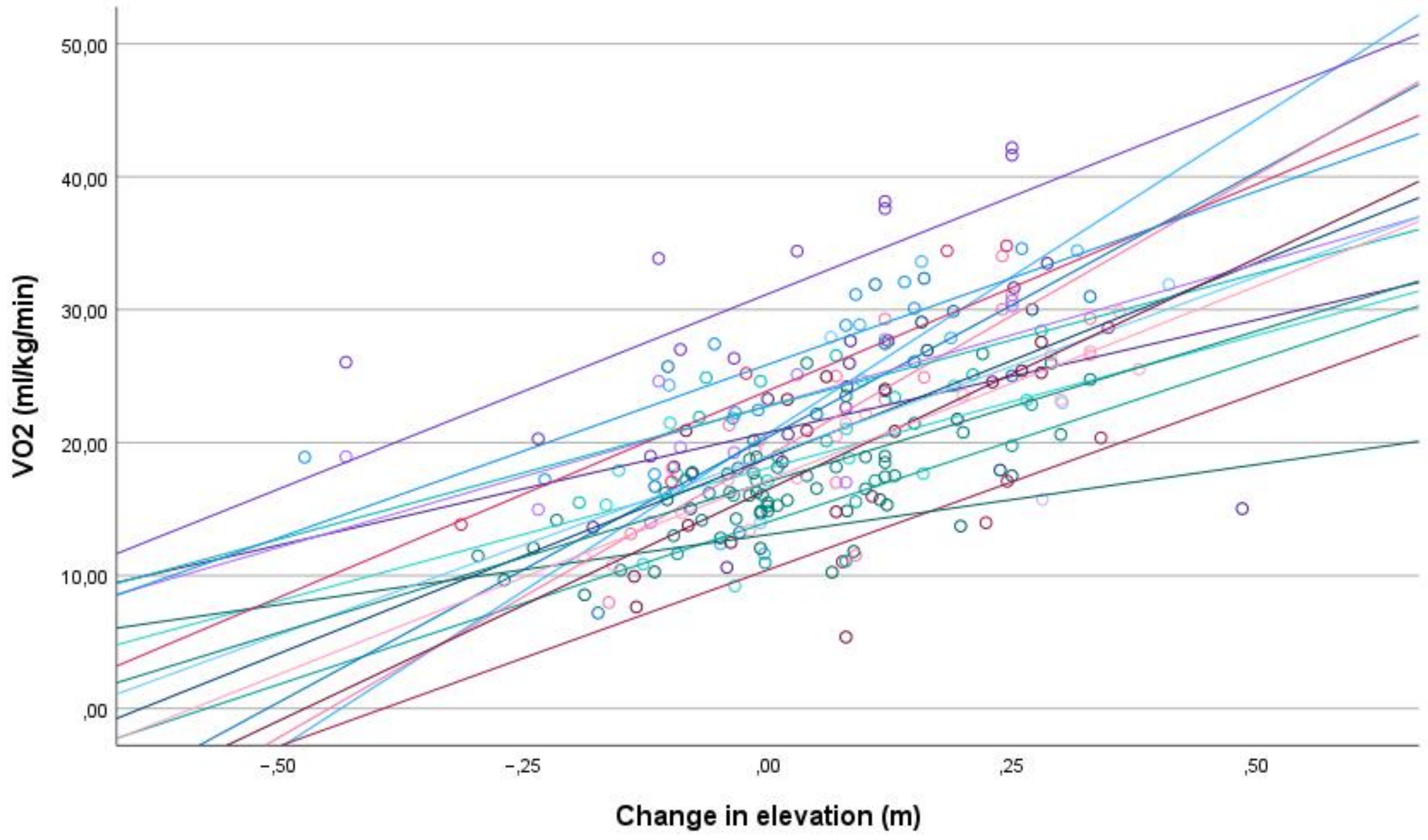

Supplementary figure 1. Scatterplot with individual fit lines showing the association between change in elevation and oxygen consumption averaged over a) 10 seconds (standardized β =0.402, p= < 0.001, 1345 observations), b) 30 seconds (standardized β =0.507, p=<0.001, 457 observations) and c) 60 seconds (standardized β =0.529, p<0.001, 233 observations) during 19 e-bike commutes.
